# Exploration of the Potential Mechanisms of Wumei Pill for the Treatment of Ulcerative Colitis by Network Pharmacology

**DOI:** 10.1155/2021/4227668

**Published:** 2021-12-21

**Authors:** Jinke Huang, Yijun Zheng, Jinxin Ma, Jing Ma, Mengxiong Lu, Xiangxue Ma, Fengyun Wang, Xudong Tang

**Affiliations:** ^1^Department of Gastroenterology, Xiyuan Hospital of China Academy of Chinese Medical Sciences, Beijing, China; ^2^Department of Gastroenterology, Peking University Traditional Chinese Medicine Clinical Medical School (Xiyuan), Beijing, China; ^3^China Academy of Chinese Medical Sciences, Beijing, China

## Abstract

**Background:**

Wumei pill (WMP) has a long history of colitis treatment in China, but the protective mechanisms have not been elucidated. To uncover the potential mechanisms of WMP against ulcerative colitis (UC), the network pharmacology approach was utilized in this study.

**Methods:**

Public databases were utilized to identify the potential targets of WMP and genes related to UC. Based on the identified overlapping common targets, drug-ingredient-target gene network, Gene Ontology (GO) analysis, Kyoto Encyclopedia of Genes and Genomes (KEGG) analysis, and protein-protein interaction (PPI) analysis were conducted. Molecular docking was carried out to verify the selected key active ingredients and core targets.

**Results:**

129 active ingredients and 622 target genes were obtained. The drug-ingredient-target gene network revealed 52 active ingredients of WMP acting on 73 targets related to UC. GO analysis revealed that biological processes were mainly associated with oxidative stress, such as, reactive oxygen species metabolic processes, response to oxidative stress, cellular response to oxidative stress, response to reactive oxygen species, and regulation of reactive oxygen species metabolic processes. KEGG analysis revealed that the immune- and inflammation-related pathways, tumor-related signaling pathways, and microbial infection-related signaling pathways were the most significant. PPI network identified 13 core target genes. The molecular docking results indicated the formation of stable bonds between the active ingredients and core target genes.

**Conclusions:**

The approach of network pharmacology reveals the key ingredients, potential core targets, and biological process of WMP in the treatment of UC. The mechanisms of action of WMP involve anti-inflammation, antioxidation, and modulation of immunity, which provides evidence for the therapeutic role of WMP in UC.

## 1. Introduction

Ulcerative colitis (UC) is an inflammatory bowel disease characterized by relapsing and remitting mucosal inflammation [1]. Globally, the incidence of UC is increasing, with 24.3/100,000 in Northern Europe [2]. UC results in a heavy socioeconomic burden, with annual costs of $8.1–$14.9 billion in the United States and €12.5–29.1 billion in Europe [3]. UC is a dynamic disease with severity that can change over time [4], and the etiologies have not been fully elucidated. The therapeutic armamentarium for UC is expanding [1]; however, common therapies have been reported to be far less effective than ideal [5].

Chinese herbal medicine (CHM) has been widely used in colitis treatment in China, with potential benefits including high efficacy, safety, and relatively low economic costs [6]. Since CHM prescriptions contain complex compounds, they often have various multitargeted and synergistic effects. Wumei pill (WMP) originated from Shang Han Za Bing Lun (200–210, AD) with10 herbs (Wumei (Mume Fructus), Xixin (Asari Radix Et Rhizoma), Guizhi (Cinnamomi Ramulus), Huanglian (Coptidis Rhizoma), Huangbai (Coptidis Rhizoma), Danggui (Angelicae Sinensis Radix), Rensen (Panax Ginseng C. A. Mey.), Huajiao (Zanthoxyli Pericarpium), Ganjiang (Zingiberis Rhizoma), and Fuzi (Aconiti Lateralis Radix Praeparata)) and has been widely used to treat UC in clinical practice. Evidence from meta-analysis suggested that the clinical efficacy of combined use of WMP with conventional medicine is better than that of conventional medicine alone [7]. Despite its clinical effectiveness, the potential mechanisms of WMP on UC are not yet explained.

**Figure 1:**
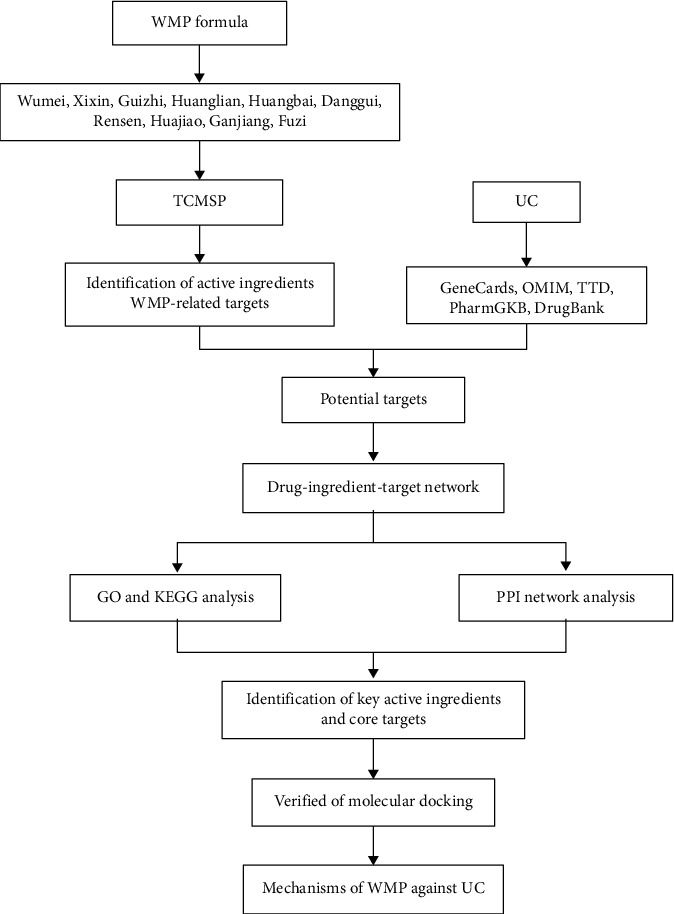
Workflow of the study.

Network pharmacology is a priori analytical approach that combines system network analysis with pharmacology and can efficiently elucidate the relationship between drugs, compounds, diseases, and targets [8, 9]. Thereby, network pharmacology was utilized to explore the potential mechanisms of WMP against UC in this study.  Figure 1 illustrates the workflow.

## 2. Methods

### 2.1. Active Ingredient and Target Gene Screening

A systematic search was conducted in Traditional Chinese Medicine Systems Pharmacology (TCMSP) database (https://tcmspw.com/tcmsp.php) [10] to obtain ingredients of WMP. The oral bioavailability ≥ 30% and drug-likeness ≥ 0.18 were established as screening criteria [10].

Target genes corresponding to WMP that obtained from TCMSP were imported into UniProt (https://www.uniprot.org/) [11] to obtain standard gene symbols.

### 2.2. Acquisition of UC-Related Target Genes

Targets related to UC were retrieved from the follow public databases: GeneCards database (https://www.genecards.org/), PharmGKB database (https://www.pharmgkb.org/), OMIM database (https://omim.org/), TTD database (http://bidd.nus.edu.sg/group/cjttd/), and DrugBank database (https://www.drugbank.ca/). Targets from GeneCards with the relevance score ≥ 5 were screened out [12].

### 2.3. Drug-Ingredient-Target Network Construction

Intersections of target genes for drugs and diseases were obtained through a Venn diagram, and the overlapping genes were considered potential targets of WMP for UC. Cytoscape 3.7.2 software was utilized to establish the drug-ingredient-target network [13].

### 2.4. Analysis of GO and KEGG Pathway

To further investigate the biological characteristics of WMP on UC, analysis of GO and KEGG pathway was carried out using the clusterProfiler package [14] in R 4.0.5 software. *P* < 0.05 was considered statistically significant.

### 2.5. PPI Network Construction and Core Gene Screening

PPI analysis was carried out in STRING database (https://string-db.org/) [15] and visualized with Cytoscape 3.7.2 software. The minimum required interaction score was set as the “highest confidence (0.400).” The core genes were screened through Cytoscape plugin cytoHubba [16] by calculate betweenness centrality, closeness centrality, degree centrality, eigenvector centrality, network centrality, and local average connectivity. The target nodes with all six parameters above the corresponding median value in the PPI network were reserved to build a new PPI network for core gene screening.

### 2.6. Verification through Molecular Docking

Molecular docking was performed to validate the compound-target associations. Structures of compounds were downloaded from the PubChem (https://pubchem.ncbi.nlm.nih.gov/) [17], and the macromolecular protein target receptors were downloaded from the RCSB PDB (http://www.rcsb.org/) [18]. Molecular docking was performed by AutoDock Vina [19]. The value of the Vina score less than “−5” indicates a good binding interaction between the compound and target [20], and the results were visualized using PyMOL [21].

## 3. Results

### 3.1. Active Ingredients and Target Genes

From the TCMSP database, a total of 129 active ingredients were identified, including 8 ingredients of Wumei, 8 of Xixin, 7 of Guizhi, 14 of Huanglian, 37 of Huangbai, 2 of Dangui, 22 of Rensen, 5 of Huajiao, 5 of Ganjiang, and 21 of Fuzi. Moreover, 1927 target genes were obtained and subsequently uploaded to UniProt to obtain standard gene symbols. After eliminating the redundancy, 1710 target genes were identified finally. Details of the 129 active ingredients and the 1710 target can be found in supplementary A and supplementary B, respectively.

### 3.2. Target Genes Related to UC

In total, 5273 target genes were identified, including 549 from the GeneCards, 3 from OMIM, 15 from PharmGKB, 15 from TTD, and 189 from DrugBank. After eliminating the redundancy, 622 target genes were obtained finally. Details of the 622 target genes can be found in supplementary C.

### 3.3. Construction of Drug-Ingredient-Target Gene Network

**Figure 2:**
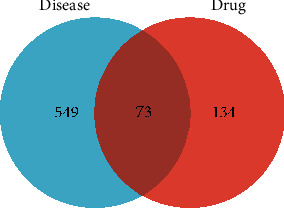
Venn diagram of targets from WMP and UC.

**Figure 3:**
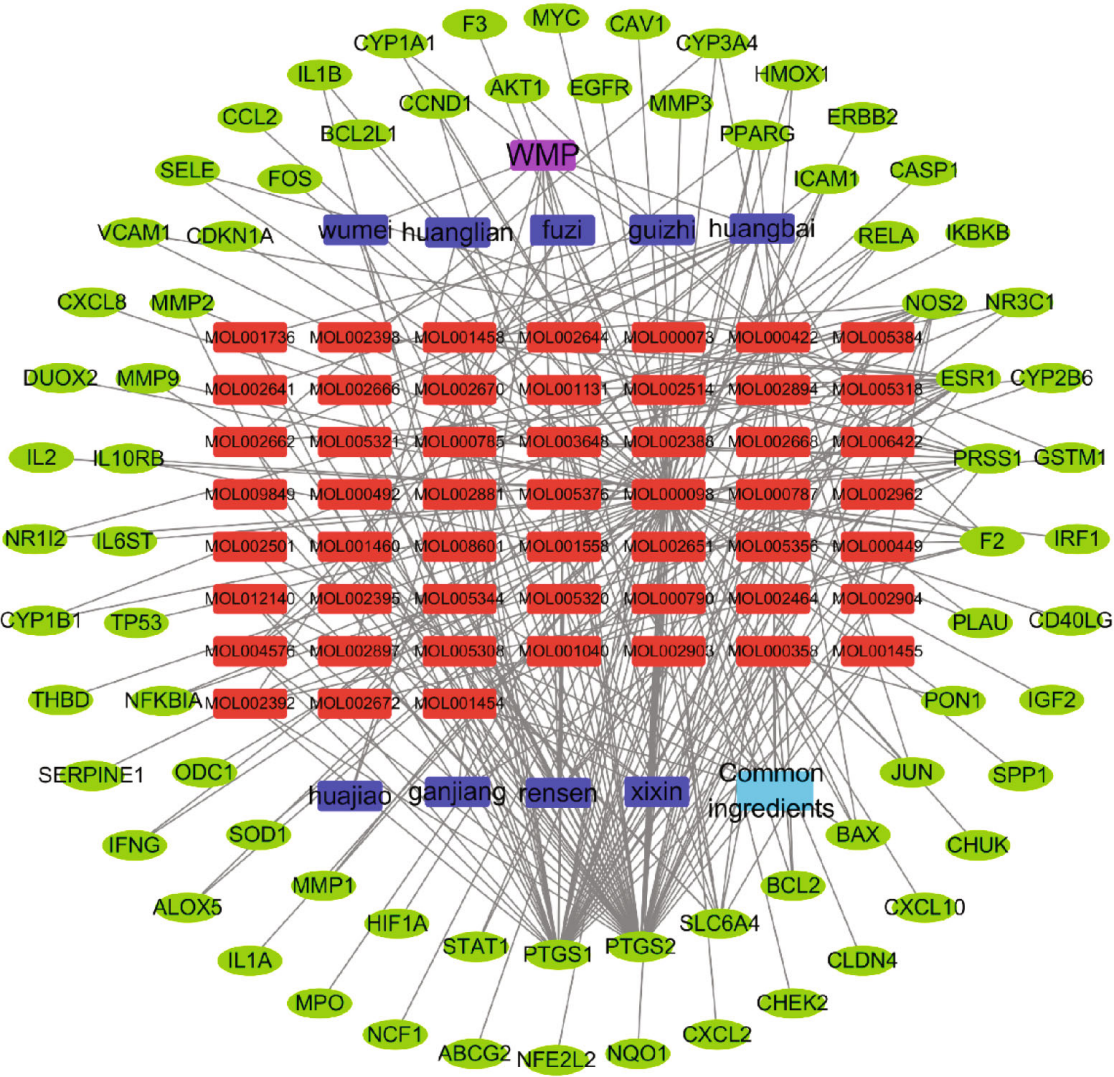
Drug-ingredient-target gene network of WMP.

A Venn diagram (Figure 2) identified 73 overlapping genes. Subsequently, a drug-ingredient-target gene network was constructed, which included 52 ingredients, 73 target genes, 136 nodes, and 308 edges (Figure 3).

### 3.4. GO and KEGG Pathway Analyses

**Figure 4:**
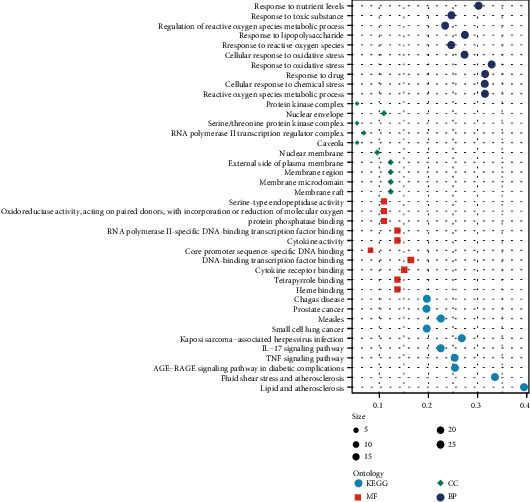
Results of GO and KEGG pathway analyses.

The results of GO and KEGG pathway analyses were visualized in Figure 4. Based on the biological processes, the targets were mainly enriched in reactive oxygen species metabolic process, cellular response to chemical stress, response to drug, response to oxidative stress, cellular response to oxidative stress, response to reactive oxygen species, response to lipopolysaccharide, regulation of reactive oxygen species metabolic process, response to toxic substance, and response to nutrient levels. The top 10 significantly enriched pathways contained pathways in cancer, lipid and atherosclerosis, fluid shear stress and atherosclerosis, AGE-RAGE pathways, TNF pathways, IL-17 pathways, and microbial infection-related pathways.

### 3.5. PPI Network and Core Gene Analysis

**Figure 5:**
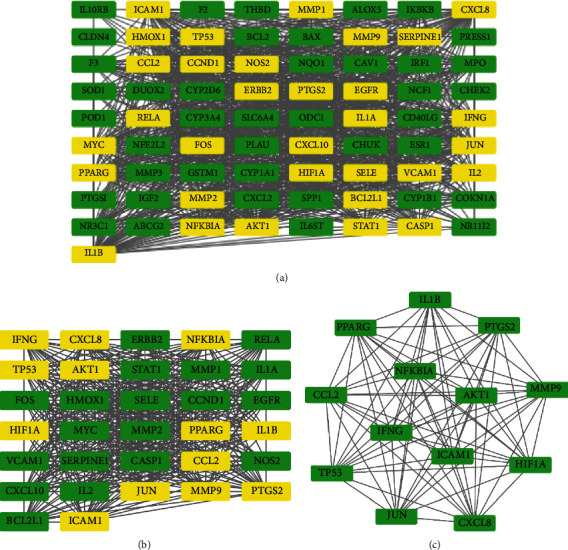
Process of topological screening for the PPI network. (a) PPI network from STRING visualized with Cytoscape. (b) PPI network of more significant proteins extracted from (a) by filtering 6 parameters: BC, CC, DC, EC, NC, and LAC. (c) Core PPI network of core targets extracted from (b).

A PPI network with 73 nodes and 2150 edges was obtained in STRING. Through Cytoscape plugin cytoHubba, the results of the first screening found 32 nodes and 440 edges. For the second screening, a dense region network with 13 nodes and 78 edges was obtained. The 13 core target genes included CCL2, HIF1A, JUN, NFKBIA, MMP9, CXCL8, IL1B, TP53, AKT1, IFNG, PPARG, PTGS2, and ICAM1. Details are presented in Figure 5.

### 3.6. Molecular Docking Analysis

**Figure 6:**
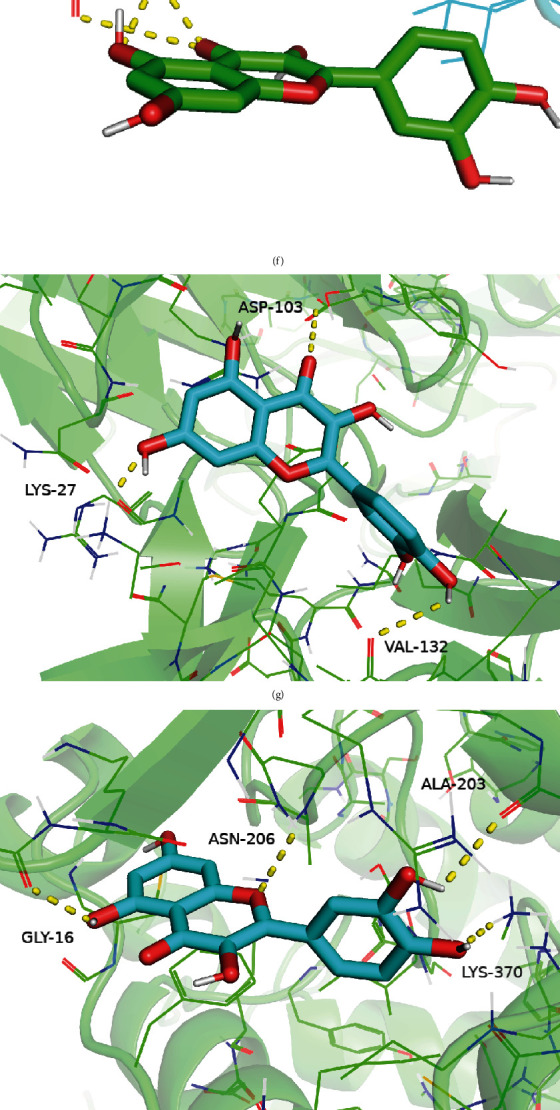
Molecular docking models. (a) CCL2, (b) HIF1A, (c) JUN, (d) NFKBIA, (e) MMP9, (f) CXCL8, (g) IL1B, (h) TP53, (i) AKT1, (j) IFNG, (k) PPARG, (l) PTGS2, and (m) CAM1.

With molecular docking, the results showed that all values of the Vina score less than “−5,” suggesting that the key active ingredients have good binding ability with the core target genes (Figure 6, Table 1).

## 4. Discussion

UC is an urgent global public health concern. Although conventional drugs such as 5-aminosalicylates, hormones, and immunosuppressive agents have played a good role in the treatment of UC, they still failed to achieve ideal effects and were often accompanied by side effects [5]. In China, CHM prescriptions have been widely used to treat UC and show beneficial preventive and therapeutic effects [6]. However, complex components and targets also pose a challenge for mechanistic researches [22]. Through the method of network pharmacology, the targets and specific mechanisms of CHM prescriptions on diseases can be more clearly defined, which is of great value for the research and development of natural medicines. Therefore, in this study, network pharmacology was applied to uncover the mechanisms of WMP against UC. The results suggested that 52 active ingredients of WMP act on 73 UC-related targets. Further analysis revealed that multiple biological processes were involved, such as reactive oxygen species metabolic processes, cellular responses to chemical stress, and responses to oxidative stress. And WMP might have effects on the outcomes of UC through the IL-17 pathway, TNF pathway, AGE-RAGE pathway, and cancer-related pathways.

For the targets of WMP on UC, GO analysis showed that the enriched biological processes were mainly focused on oxidative stress. Oxidative stress stems from the altered balance between reactive oxygen species production and the ability to rapidly detoxify reactive intermediates [23], and excess ROS can destroy oxidizable biomolecules and form lipid peroxidation products, which in turn disrupt cell membrane function and structure [24, 25]. Thus, oxidative stress is one of the triggering factors for UC development [26, 27]. Furthermore, increased oxidative stress is associated with mucosal inflammation in UC, and it may be a contributing factor to the progression to malignancy associated with this disease [23]. Importantly, inhibition of oxidative stress in colon tissues has been reported to have beneficial effects on lowering intestinal inflammation and altering the gut microbiome diversity [28]. Results of this study revealed that WMP is mainly involved in regulating oxidative stress; thus, it can be considered a potential option for the UC treatment. For KEGG analysis, the most significant pathways are all associated with oxidative stress, namely, pathways in cancer [29], lipid and atherosclerosis [30], and fluid shear stress and atherosclerosis [31]; AGE-RAGE pathway [32]; TNF pathway [33]; IL-17 pathway [34]; and microbial infection-related pathways [35]. Moreover, most of the signaling pathways are also involved in immune and inflammatory responses, which are closely related to UC. For example, IL-17, which is secreted by T17 cells, acts as a key mediator in the pathogenesis of intestinal inflammation [36]. Furthermore, IL-17 can be promoted by IL-23 to increase, thus forming the IL-23/17 axis to amplify the inflammatory response [37]. By producing antimicrobial peptides, IL-17A can also be involved in amplification of inflammatory responses and regulation of mucosal barrier function [38].

PPI network was constructed to explore the key active ingredients and core targets. The results revealed that quercetin, kaempferol, ginsenoside rh2, frutinone A, and dianthramine were the key active ingredients of WMP in the treatment of UC. Previous studies have shown that these key active ingredients have antioxidant and anti-inflammatory effects and can modulate the immune system at the molecular level [39]. It is reported that quercetin administration can reduce the levels of TNF-*α* and lipocalin-2 mRNA and enhance the expression of Slip protein, which in turn inhibits inflammation in UC organoids [40]. Kanagol has the property of protecting endothelial cells from oxidative damage, which in turn can be used to treat inflammatory diseases [41]. Similarly, ginsenosides have benefits in enhancing immunity and can be involved in regulatory processes of inflammation by affecting the immune system [42]. Moreover, CCL2, HIF1A, JUN, NFKBIA, MMP9, CXCL8, IL1B, TP53, AKT1, IFNG, PPARG, PTGS2, and ICAM1 were identified as core targets. CCL2 and CXCL8 belong to a family of chemokines, which are mainly involved in immunoregulatory and inflammatory processes [43]. HIF1A acts as a master regulator of cellular and systemic homeostatic responses to hypoxia and has a regulatory role in intestinal mucosal inflammation in UC patients [44]. For JUN, it has been confirmed to improve gastrointestinal mucosal conditions in UC patients by modulating JUN-related pathways [45]. NFKBIA encodes a member of the NF-*κ*B inhibitor family, which plays a key role in inflammation, oxidative stress, and immunity [46]. Expression of MMP9 is elevated in UC, and MMP9 inhibitors are a promising therapeutic strategy for the treatment of UC patients with MMP9 upregulation [47]. IL-1B is a member of the interleukin 1 cytokine family, and UC is largely the result of cytokines such as IL-1B promoting inflammatory responses [48]. TP53 is a tumor suppressor protein; a previous study indicated that alterations in TP53 may be an early biomarker of a progressor colon and that TP53 is accumulated early in UC-related carcinogenesis [48]. Similarly, AKT1 has been confirmed to have a relationship between UC and colon adenocarcinoma [49]. IFNG encodes a soluble cytokine, and it can be used as a blood marker for antitumor necrosis factor therapy in IBD patients [50]. PPARG is closely related to oxidative stress, and partial PPAR-*γ* agonists may be a new target for UC treatment [51]. PTGS is responsible for prostaglandin biosynthesis involved in inflammation and mitosis, and it has been identified as crucial genes related to UC [52]. Similarly, ICAM1, which is typically expressed on endothelial cells and cells of the immune system, has been also identified as crucial genes related to UC by bioinformatics analysis [52].

Furthermore, the molecular docking validation results showed that the key active ingredients had good binding ability to the core target genes, indicating that the potential mechanism of WMP for UC treatment revealed by the network pharmacology approach is reasonable.

Limitations must be acknowledged. First, the use of network pharmacology to reveal the mechanism of WMP in the treatment of UC is an in silico prediction method; thus, further in vivo experimental validation is required to support the reliability of the prediction results. Furthermore, the application of network pharmacology in CHM research is only in its infancy; further integration of multiple disciplines such as bioinformatics, computer science, and pharmacology needs to be promoted to improve the scientificity of research methods.

## 5. Conclusion

The approach of network pharmacology reveals the key ingredients, potential core targets, and biological process of WMP in the treatment of UC. The mechanisms of action of WMP involve anti-inflammation, antioxidation, and modulation of immunity, which provides evidence for the therapeutic role of WMP in UC.

## Figures and Tables

**Table 1:** Results of molecular docking.

Query	Core genes	PDB ID	Ingredients	Affinity (kcal/mol)
1	CCL2	2nz1	Quercetin	-8.1
2	HIF1A	3hqu	Quercetin	-8.1
3	JUN	1jnm	Quercetin	-8.0
4	NFKBIA	1nfi	Quercetin	-8.8
5	MMP9	1itv	Quercetin	-8.5
6	CXCL8	4xdx	Quercetin	-7.1
7	IL1B	5mvz	Quercetin	-8.0
8	TP53	3tg5	Quercetin	-9.9
9	AKT1	6hhg	Kaempferol	-9.2
10	IFNG	1fyh	Ginsenoside rh2	-9.1
11	PPARG	4a4w	Frutinone A	-10.0
12	PTGS2	5kir	Dianthramine	-8.5
13	ICAM1	5mza	Kaempferol	-6.9

## Data Availability

All data obtained or analyzed during this work are included within the article.

## Abbreviations

WMP: Wumei pill
UC: Ulcerative colitis
CHM: Chinese herbal medicine
GO: Gene Ontology
KEGG: Kyoto Encyclopedia of Genes and Genomes
PPI: Protein-protein interaction.

## Supplementary Materials

Supplementary Materials The 129 active ingredients of WMP and the 1710 target can be found in supplementary A and supplementary B, respectively. Target genes related to UC can be found in supplementary C.
